# May-Thurner Syndrome as a Rare Cause of Paradoxical Embolism in a Patient with Patent Foramen Ovale

**DOI:** 10.1155/2018/3625401

**Published:** 2018-07-02

**Authors:** Dominika M. Zoltowska, Guramrinder Thind, Yashwant Agrawal, Vishal Gupta, Jagadeesh Kumar Kalavakunta

**Affiliations:** ^1^Department of Internal Medicine, Western Michigan University Homer Stryker M.D. School of Medicine, 300 Portage Street, Kalamazoo, MI 49007, USA; ^2^Department of Cardiology, St. Joseph Mercy Oakland Hospital, 44405 Woodward Ave, Pontiac, MI 48341, USA; ^3^Department of Cardiology, Michigan State University and Borgess Medical Center, 1521 Gull Rd, Kalamazoo, MI 49048, USA

## Abstract

May-Thurner syndrome is an underrecognized anatomical variant that can lead to increased propensity for venous thrombosis in the lower extremities. We present a case of a 67-year-old female who presented with transient ischemic attack. Initial workup including CT scan of the head, MRI scan of the head, and magnetic resonance angiogram of the head and neck was unremarkable. A transthoracic echocardiogram with bubble study was also normal. Subsequently, a transesophageal echocardiogram was performed that revealed a patent foramen ovale with right-to-left shunt. Lower extremity duplex venous ultrasound showed no evidence of deep vein thrombosis. However, magnetic resonance venogram of the pelvis showed compression of the left common iliac vein just after its origin suggestive of May-Thurner syndrome. Hence, May-Thurner syndrome was recognized as the probable source of paradoxical embolism causing transient ischemic attack in this patient.

## 1. Background

May-Thurner syndrome (MTS) or iliocaval compression is an uncommon anatomical variant characterized by external compression of the left common iliac vein by the right common iliac artery against the lumbar vertebra [1]. MTS commonly presents as deep venous thrombosis (DVT) or with clinical features of chronic venous insufficiency. However, there has been growing evidence associating MTS with stroke or transient ischemic attack (TIA) caused by paradoxical embolism in the setting of patent foramen ovale (PFO). Recognition and follow-up of MTS in these scenarios are important.

## 2. Case

A 67-year-old female with past medical history of congenital deafness presented to the emergency room with complaints of right-sided facial droop and right upper extremity weakness, tingling, and numbness. These symptoms were sudden in onset and lasted for a few minutes. Symptoms had completely resolved at the time of presentation. She did not have a history of any atherosclerotic risk factor including hypertension, diabetes, or hypercholesterolemia. The initial set of vital signs were normal; routine laboratory tests including complete blood count and basic metabolic panel were unremarkable. A computed tomography (CT) of the head without contrast as well as a magnetic resonance imaging (MRI) of the brain with and without contrast did not show any acute intracranial hemorrhage or infarction.

**Figure 1:**
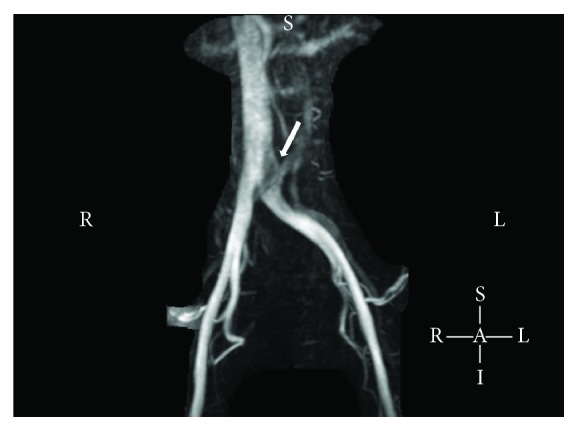
Magnetic resonance venogram of the pelvis showing compression of the left common iliac vein (arrow) just after its origin suggestive of May-Thurner syndrome.

The patient was diagnosed with TIA, and further investigations were planned to determine the etiology. A magnetic resonance angiogram (MRA) of the head and neck with and without contrast did not show any arterial flow limiting stenosis or occlusion. A transthoracic echocardiogram (TTE) with bubble study using agitated normal saline contrast was performed and was found to be normal. Patient's heart rhythm was monitored with continuous cardiac monitoring, and no arrhythmias were noted during her stay at the hospital. At this point, the patient was identified as having cryptogenic TIA, having failed to determine the precise etiology from routine workup. Patient was started on aspirin therapy and discharged from the hospital on day 3 with further outpatient workup planned. Outpatient workup for hypercoagulability showed a high factor VIII activity of 153%, which potentially put her at increased risk of venous thromboembolism. However, this test was performed just one week after the thrombotic event and was hence difficult to interpret. Subsequently, a transesophageal echocardiogram (TEE) was performed that revealed a patent foramen ovale with right-to-left shunt. This raised the concern for paradoxical embolism as the cause of patient's TIA. Lower extremity duplex venous ultrasound showed no evidence of deep vein thrombosis. However, magnetic resonance venogram (MRV) of pelvis showed compression of the left common iliac vein just after its origin, which was suggestive of May-Thurner syndrome (Figure 1). There was no evidence of venous thrombosis on the MRV. May-Thurner syndrome was recognized as the probable source of paradoxical embolism causing TIA in the patient. The patient was eventually referred for percutaneous PFO repair, which was performed without any complications. The patient had been regularly followed yearly at the cardiology clinic for 5 years now. She remains in good health with no further episodes of TIA.

## 3. Discussion

Cryptogenic stroke or TIA is a clinical entity that describes stroke or TIA in a patient where the initial routine workup is negative. Hence, no obvious evidence of large artery atherosclerosis, cardioembolism, or small artery disease is discovered on initial evaluation. Up to 40% of ischemic strokes are eventually classified as cryptogenic [2, 3]. Most cryptogenic strokes/TIAs are thought to be thromboembolic. Paradoxical embolism originating from a venous source has been identified as a potential cause of stroke/TIA in patients with PFO. The association of paradoxical embolism and cryptogenic stroke/TIA is backed up by the fact that a higher prevalence of PFO has been documented in patients with cryptogenic stroke (up to 56%) as compared to the general population (20 to 26%) [4–7]. These patients usually have a lower prevalence of cardiovascular risk factors including hypertension, hypercholesterolemia, and diabetes [8].

In patients presenting with stroke or TIA where the initial workup is negative, further workup should include echocardiogram with bubble studies to look for a PFO. TTE with bubble study is the more popular initial test used to diagnose PFO. Although it is highly specific, it has low sensitivity when compared to TEE. In a meta-analysis of 13 studies performed by Mojadidi et al., the accuracy of TTE to detect intracardiac right-to-left shunt was compared to TEE as the reference [9]. The weighted sensitivity and specificity for TTE were found to be 46% and 99%, respectively. Hence, although TTE is highly specific for the detection of right-to-left intracardiac shunts, a negative test with TTE does not rule out the presence of a PFO and the same should be confirmed by a TEE.

When a PFO is detected during the workup of stroke or TIA, further investigations should be performed to look for venous thrombosis as a source of paradoxical embolism. Lower extremity duplex venous ultrasound is often the initial test performed in these patients to exclude lower extremity DVT. However, pelvic and iliac vein thrombosis can be missed with ultrasound because of overlying bowel gas [10]. Furthermore, ultrasound may not detect May-Thurner syndrome. Although no specific guidelines exist, magnetic resonance venography can be performed in these patients for better visualization of pelvic and iliac vein anatomy. In a retrospective analysis by Osgood et al., 50 patients with cryptogenic stroke and PFO underwent contrast-enhanced pelvic MRV. Out of these, four patients (8%) were found to have pelvic vein thrombosis and five patients (10%) were found to have May-Thurner syndrome [11].

MTS anatomy is characterized by partial or complete impedance to the common iliac vein outflow with subsequent obstruction and thrombus formation [1]. Most of these patients are asymptomatic; however, they can have clinical manifestations secondary to thrombosis or mechanical venous occlusion. MTS can present as acute DVT, venous claudication, skin changes due to chronic venous insufficiency, or rarely pulmonary embolism. In patients with MTS, stasis of blood due to occlusion serves as a potential source of small clots that can embolize. It has been hypothesized that these clots are often too small to cause clinically significant pulmonary embolism. However, in patients with PFO, paradoxical embolism can cause a stroke or TIA [12].

As mentioned before, Doppler ultrasound can miss abnormalities in iliac veins and is hence not the ideal imaging modality for the diagnosis of MTS. MRV can provide definitive evidence of May-Thurner syndrome anatomy. If MTS is associated with active thrombosis or if sequelae of chronic venous hypertension are present, these patients can be treated with angioplasty and stenting [1]. Further anatomic detailing to assist with stent deployment can be achieved with intravascular ultrasound or invasive venography.

The issue of PFO closure in patients with cryptogenic stroke/TIA is controversial. Lack of ability to objectively identify paradoxical embolism as the cause of stroke/TIA and questionable efficacy of closure devices to prevent paradoxical embolism are some of the major concerns. The latest American Academy of Neurology guidelines recommend against routine PFO closure in patients with cryptogenic stroke without concomitant DVT [13]. However, these guidelines advocate consideration of PFO closure in patients with recurrent cryptogenic strokes. Furthermore, the American Heart Association/American Stroke Association guidelines advocate consideration of PFO closure in patients with cryptogenic stroke who also have DVT [14]. As highlighted before, May-Thurner syndrome is a potential source of venous thromboembolism. Hence, these patients with cryptogenic stroke may benefit from PFO closure. However, more data is needed before any strong recommendations can be made in this regard.
